# Site-specific chromatin immunoprecipitation: a selective method to individually analyze neighboring transcription factor binding sites in vivo

**DOI:** 10.1186/1756-0500-5-109

**Published:** 2012-02-20

**Authors:** Ronaldo Schuch, Konstantin Agelopoulos, Anna Neumann, Burkhard Brandt, Horst Bürger, Eberhard Korsching

**Affiliations:** 1 Institute of Bioinformatics, University Hospital of Muenster, Muenster, Germany; 2 Department of Medicine A, Hematology and Oncology, University Hospital of Muenster, Muenster, Germany; 3 Institute of Clinical Chemistry, University Medical Center Schleswig-Holstein, Kiel, Germany; 4 Institute of Pathology, Cooperative Breast Center, Paderborn, Germany; 5 Institute of Bioinformatics, University Hospital of Muenster, Niels-Stensen-Str. 12, 48149 Muenster, Germany

## Abstract

**Background:**

Transcription factors (TFs) and their binding sites (TFBSs) play a central role in the regulation of gene expression. It is therefore vital to know how the allocation pattern of TFBSs affects the functioning of any particular gene in vivo. A widely used method to analyze TFBSs in vivo is the chromatin immunoprecipitation (ChIP). However, this method in its present state does not enable the individual investigation of densely arranged TFBSs due to the underlying unspecific DNA fragmentation technique. This study describes a site-specific ChIP which aggregates the benefits of both EMSA and in vivo footprinting in only one assay, thereby allowing the individual detection and analysis of single binding motifs.

**Findings:**

The standard ChIP protocol was modified by replacing the conventional DNA fragmentation, i. e. via sonication or undirected enzymatic digestion (by MNase), through a sequence specific enzymatic digestion step. This alteration enables the specific immunoprecipitation and individual examination of occupied sites, even in a complex system of adjacent binding motifs in vivo. Immunoprecipitated chromatin was analyzed by PCR using two primer sets - one for the specific detection of precipitated TFBSs and one for the validation of completeness of the enzyme digestion step. The method was established exemplary for Sp1 TFBSs within the *egfr *promoter region. Using this site-specific ChIP, we were able to confirm four previously described Sp1 binding sites within *egfr *promoter region to be occupied by Sp1 in vivo. Despite the dense arrangement of the Sp1 TFBSs the improved ChIP method was able to individually examine the allocation of all adjacent Sp1 TFBS at once. The broad applicability of this site-specific ChIP could be demonstrated by analyzing these SP1 motifs in both osteosarcoma cells and kidney carcinoma tissue.

**Conclusions:**

The ChIP technology is a powerful tool for investigating transcription factors in vivo, especially in cancer biology. The established site-specific enzyme digestion enables a reliable and individual detection option for densely arranged binding motifs in vivo not provided by e.g. EMSA or in vivo footprinting. Given the important function of transcription factors in neoplastic mechanism, our method enables a broad diversity of application options for clinical studies.

## Background

Transcription factors (TFs) are core elements of transcriptional regulation and play also an important role in the systems biology of cancer characterized by changes in the expression levels of certain genes [1]. A complex of more than 20 TFs molecules is involved in RNA polymerase II initiation of transcription in the promoter region for the majority of the genes [2]. The activity of the transcription machinery is based on the arrangement and the occupancy of transcription factor binding sites (TFBSs) along the 5'-region of the gene. Because of this dense arrangement and the necessity to analyze the individual occupancy of the TFBSs to establish regulation models, there is a strong demand for methods that enable this type of individual analysis.

So far, several methods have been developed for identification and analysis of TFBSs. A commonly used method to verify TFBSs is the electrophoretic mobility shift assay (EMSA) [3]. Indeed, even though the gel mobility shift analysis provides a fast and easy identification of which nucleotides are required for TF binding, it does not work under in vivo conditions [4]. On the other hand, the method of in vivo footprinting [5] enables the investigation of protein binding in living cells, but this technique is only capable of identifying DNA regions that are bound by protein, being not able to identify which protein is responsible for the observed footprint [4,6].

**Figure 1:**
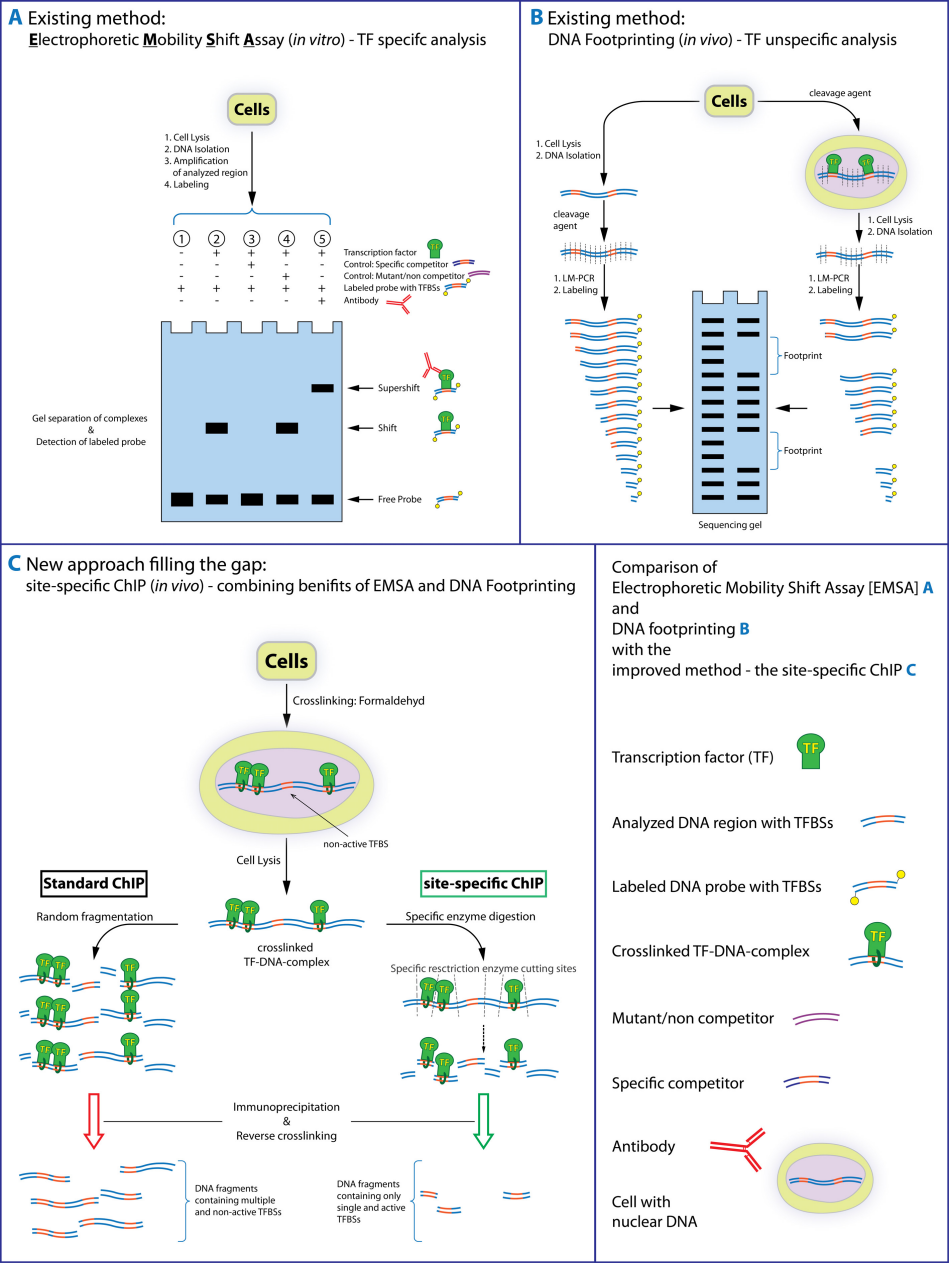
**Comparison of actual methods for TFBS analysis with the site-specific ChIP**. Principles of commonly applied techniques for TFBS analysis in comparison to the concept of the site-specific ChIP. **(A) **The Electrophoretic Mobility Shift Assay (EMSA) provides a specific identification of nucleotides sequences involved in TF binding, but does not work under in vivo conditions. **(B) **The in vivo footprinting allows the specification of which nucleotides are bound by TFs, but it is not capable to identify the protein(s) involved in binding. **(C) **Concept of the site-specific ChIP in comparison to the standard ChIP method. By replacing the traditionally used random fragmentation step with a site-specific enzyme digestion, this site-specific ChIP enables the immunoprecipitation and enrichment of single TFBSs, thus allowing a differentiation between adjacent occupied and non- occupied binding sequences in vivo.

In contrast, the chromatin immunoprecipitation (ChIP) offers a distinct advantage over EMSA and in vivo footprinting, since the ChIP technique not only specifies which nucleotides are bound, but also identifies the interacting protein(s) in the context of in vivo samples [7]. In this context we use the term in vivo to refer to any experiments performed on living cells weather within or outside a whole organism (sometimes referred to as ex vivo). Specific modifications of the ChIP assay exist to enable the analysis of mammalian tissues, thereby allowing the detection of differences in the interaction of transcription factors and promoter regions of genes in normal and neoplastic tissues [8,9]. However, the standard ChIP has its limitations. The applied fragmentation techniques (sonication or enzymatic DNA restriction by MNase digestion) are unspecific. The individual analysis of neighboring TFBSs is therefore limited, since the standard ChIP technique does not provide a DNA cleavage in specific positions flanking a sole binding motif (see Figure 1). Approaches using restriction enzyme digestion instead of the standard methods to fragment chromatin in ChIP in order to restrict analysis to particular gene regions or transcription factor binding sites has been previously described [10,11]. Nevertheless, the enzyme-based DNA fragmentation used in these procedures is applied just after immunoprecipitation or in combination with sonication. Hence, these ChIP variations neither provide an individual analysis of close neighboring TFBSs nor a differentiation between occupied or non- occupied sites. Accordingly, considering the currently available methodology and the complexity of the Protein-DNA interaction within transcriptional active gene regions, the individual analysis of neighboring TFBSs is still a challenging task.

Thus, the aim of this study was to develop and optimize a ChIP technique for the specific and individual analysis of neighboring TFBSs at once in both cell culture and tissue material.

## Findings

### Results

#### Methodological design

In order to support the individual analysis of adjacent TFBSs, we have developed an improved ChIP assay by replacing the traditionally used random fragmentation step with a site-specific enzyme digestion. This site-specific ChIP allows the immunoprecipitation and enrichment of DNA fragments containing only one TFBS, thus permitting to distinguish between adjacent occupied and non- occupied binding sequences in vivo. The principle of the site-specific ChIP and its advantages over similar procedures like EMSA and in vivo footprinting is exemplified in Figure 1.

**Figure 2:**
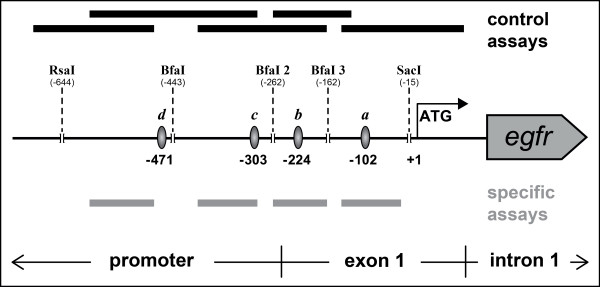
**Experimental design of the site-specific ChIP**. Schematic illustration of the strategy used for the analysis of individual TFBSs (here Sp1). Immunoprecipitation and isolation of occupied binding motifs was enabled by specific enzyme digestion on positions flanking the TFBSs. Enzyme cutting sites of the respective endonucleases (RsaI, BfaI and SacI) are depicted by dashed lines. Immunoprecipitated DNA was analyzed by PCR using two sets of primer assays; one for the specific detection of allocated Sp1 TFBSs and a control set for the determination of the efficiency of the enzyme digestion. Sp1 TFBSs within *egfr *promoter region are represented by grey oval disks, indicating their positions in relation to the translational start codon (ATG). Black bars depict the targeted *egfr *regions of the enzyme digestion control primer assays. Signals appearing in the enzyme digestion control PCR indicate a failed fragmentation. The grey bars denote location and length of PCR products of primers used for the specific detection of immunoprecipitated DNA fragments containing the targeted Sp1 binding sites (***a***, ***b***, **c**, ***d***). Signals indicate binding sites with bound TF (here Sp1).

The site-specific ChIP was established in the challenging sequence environment of the Epidermal Growth Factor Receptor gene (*egfr*) focusing on the well described transcription factor Sp1 (for experimental design see Figure 2). The *egfr *contains a GC-rich promoter region in which Sp1 has been previously described to bind to four sites [12,13]. We reanalyzed and verified these Sp1 binding sites using the TFBS prediction program MatInspector V7.4.8 [14,15]. A listing of the results obtained by the MatInspector analysis is shown on Table 1. The binding motifs are located as expected at closely neighboring positions between -471 and -88 bp upstream of the *egfr *translational start codon (Table 1 and grey oval disks, Figure 2).

**Table 1:** Results of Sp1-TFBSs verification with MatInspector

binding site	position [bp]^*‡*^		core similarity^*§*^	matrix- similarity^*$*^	sequence
				
	start	end			capitals: core sequence^*†*^
***a***	-471	-457	1,0	0,942	gtgGGGCggcgcatg
***b***	-303	-289	1,0	0,930	gcaGGGCgggaggag
***c***	-224	-210	1,0	0,956	gggGGGCggaggctg
***d***	-102	-88	0,772	0,883	gcgAGGCggggactc

^***‡ ***^Numbering of the position is relative to the *egfr *translational start codon (ATG)

^*§ *^The maximum core similarity of 1.0 is only reached when the highest conserved bases of a matrix match exactly in the sequence [14,15]

^*$ *^A perfect match to the matrix gets a score of 1.00 (each sequence position corresponds to the highest conserved nucleotide at that position in the matrix), a "good" match to the matrix usually has a similarity of > 0.80 [14,15]

^*† *^The "core sequence" of a matrix is defined as the (usually 4-5) highest conserved positions of the matrix [14,15]

Three endonucleases were utilized for the site-specific restriction: BfaI, RsaI and SacI. The immunoprecipitated sequences were analyzed by PCR using two sets of primer assays (Table 2). The first set consisted of specific primer assays targeting each binding site and was designed to measure only the specific signal of occupied TFBSs (grey bars, Figure 2). For verification purpose we designed a second set of primer assays to control the specific enzyme digestion by PCR. Each assay is spanning one enzyme cutting site (black bars, Figure 2). In the case of a successful enzyme digestion, these primer combinations should not allow the detection of the targeted sequence. The procedure was tested with the EGFR expressing osteosarcoma cell line HOS and samples of kidney carcinoma tissue. For verification purpose, the Sp1-directed site-specific ChIP was also tested on the osteosarcoma cell lines MNNG, U2-OS and SJ-SA-1 (data not shown).

**Table 2:** Primer used for analysis of site-specific ChIP products

PCR approach	Target	Primer assay	Sequence
	Sp1 binding site ***a***	Sp1-a	*For: *5'-GCACAGATTTGGCTCGACCTGGA-3' *Rev: *5'-GAGCGGGTGCCCTGAGGAGTTAATT-3'
TFBS specific	Sp1 binding site ***b***	Sp1-b	*For: *5'-TGGCCTTGGGTCCCCGCT-3' *Rev: *5'- AGGGCGGGAGGAGGAGGGAC-3
PCR	Sp1 binding site ***c***	Sp1-c	*For: *5'-TAGACGTCCGGGCAGCCCCC-3' *Rev: *5'-TCGGGACTCCGGCCGCCT-3'
	Sp1 binding site ***d***	Sp1-d	*For: *5'-AGACCGGACGACAGGCCACCT-3' *Rev: *5'-TCCCGATCAATACTGGACGGAGTCAG-3'

	RsaI cutting site	RsaI	*For: *5'-TGCCATTATCCGACGCTGGCTCTA-3' *Rev: *5'-GAGCGGGTGCCCTGAGGAGTTAATT-3'
	BfaI cutting site	BfaI	*For: *5'-GCACAGATTTGGCTCGACCTGGA-3' *Rev: *5'-AGGGCGGGAGGAGGAGGGAC-3'
enzyme digestion	BfaI 2nd cutting site	BfaI 2	*For: *5'-TGGCCTTGGGTCCCCGCT-3' *Rev: *5'-CTTGGGTCCCCGCTGCTGGTT-3'
control	BfaI 3rd cutting site	BfaI 3	*For: *5'-TAGACGTCCGGGCAGCCCCC-3' *Rev: *5'-GGTGGCCTGTCGTCCGGTCTG-3'
	SacI cutting site	SacI	*For: *5'-AGACCGGACGACAGGCCACCT-3' *Rev: *5'-CTTTTCCTCCAGAGCCCGACTCG-3'

ChIP control	GAPDH unrelated region	GAPDH	*For: *5'-GAGGAAGAGAGAGACCCTCACTG-3' *Rev: *5'-AGGGGTCTACATGGCAACTG-3'

#### Analysis of the allocation of Sp1 binding sites in EGFR expressing cells

**Figure 3:**
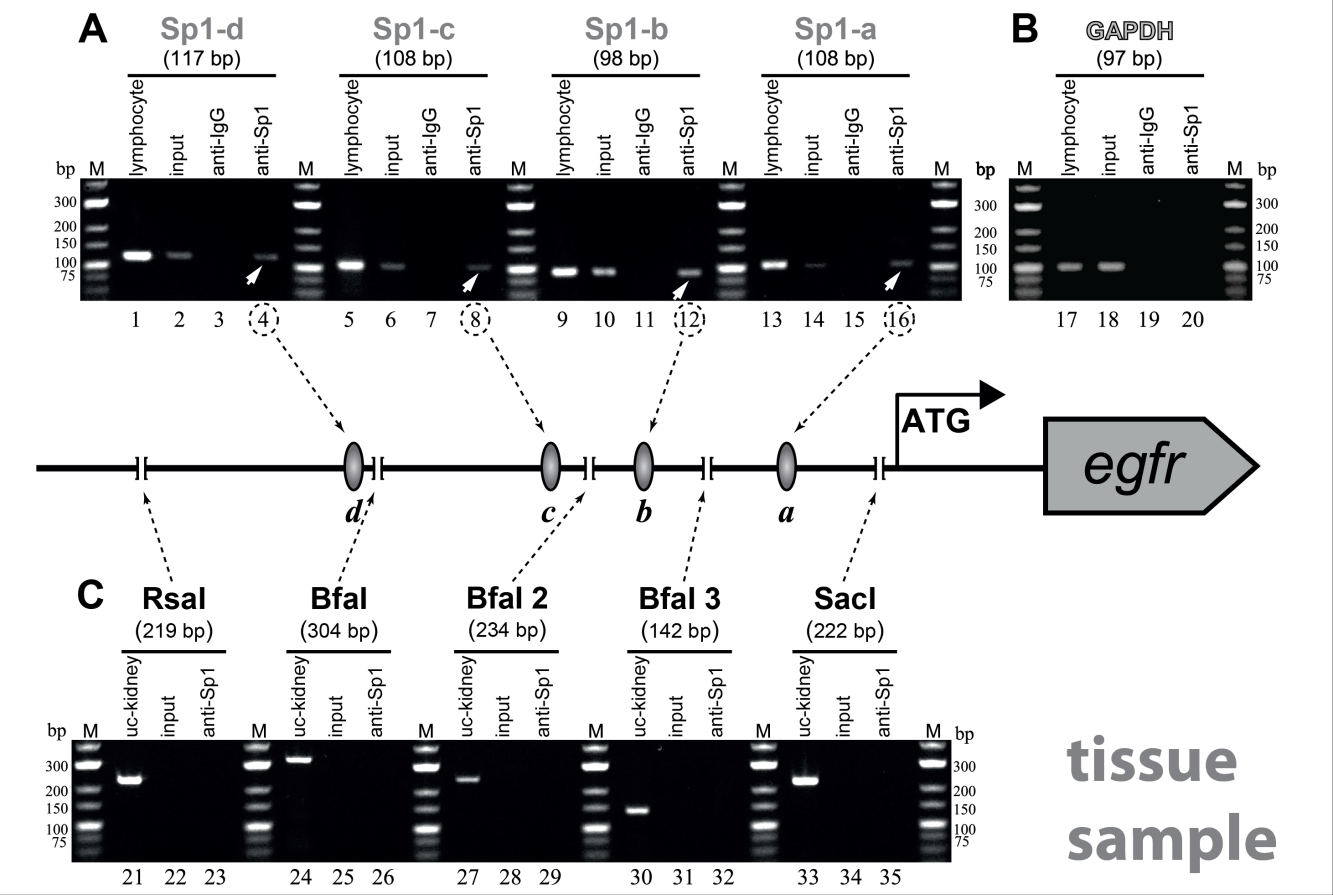
**Site-specific ChIP on cell culture extracts: Individual detection of Sp1 TFBSs within the *egfr *promoter region**. PCR-based analysis of DNA immunoprecipitated by site-specific ChIP targeting each Sp1 TFBSs within the *egfr *promoter region. Nuclei obtained from formaldehyde-fixed Osteosarcoma HOS cells were lysed, and chromatin was fragmented by specific enzyme digestion. Chromatin was immunoprecipitated either by normal rabbit IgG antibodies as a negative control or polyclonal Sp1 antiserum. Non-immunoprecipitated chromatin was used as total input control. DNAs from either the input chromatin or immunoprecipitated chromatin were subjected to PCR analysis using the Sp1 TFBSs targeting primers or restriction site flanking primers indicated in Figure 2. PCR of input DNA shows equivalent starting material for the assay. As a negative control, primers amplifying a region within the 3'-UTR of the GAPDH gene were used. In the center of the image, the *egfr *promoter region with all investigated Sp1 TFBSs and enzyme cleavage sites is shown. Dashed arrows point to the target regions of the respective PCR assay. **(A) **Lanes 1-16 show PCR products of specific assays for bound TF (Sp1) ***a ***(Sp1-a), ***b ***(Sp1-b), ***c ***(Sp1-c) und ***d ***(Sp1-d) (c.f. Figure 2 grey bars). White arrows depict the bound Sp1 binding sites. Templates order: PCR positive control (lymphocyte DNA), ChIP input control DNA, DNA immunoprecipitated with anti-IgG (IP negative control), DNA immunoprecipitated with anti-Sp1. **(B) **Lanes 17-20 show PCR products of the GAPDH control assay. **(C) **Lanes 21-35 show PCR products of primer assays for enzyme digestion control (c.f. Figure 2 black bars). Templates order: uncleaved HOS DNA from whole cell lysate (uc-HOS: PCR positive control), DNA samples immunoprecipitated with anti-IgG, DNA immunoprecipitated with anti-Sp1 antibody.

The occupation of the four known Sp1 sites was clearly confirmed in all analyzed osteosarcoma cell lines by application of the site-specific ChIP, as shown in Figure 3 for the case of the HOS cell line. All Sp1 binding sites targeting primer assays produced amplicons of the expected lengths when DNA immunoprecipitated with anti-Sp1 antibody was used as template (white arrows, Figure 3A). Moreover, PCR analysis of the input control and the anti-IgG ChIP-DNA was negative and confirmed the specificity of the site-specific ChIP (Figure 3A). The control using GAPDH primer resulted in amplification of the targeted region only when the input control was used as template, showing the accuracy of the site-specific ChIP (Figure 3B). The control of the enzymatic digestion using primers flanking enzyme cutting positions showed no amplification of the targeted sequence when DNA immunoprecipitated with anti-Sp1 antibody was used as template, proving that the DNA was cleaved at the intended sites (Figure 3C). In contrast, the same reactions using uncleaved HOS DNA of the same lysate as template produced the expected amplicons encompassing the enzyme cutting position (Figure 3C).

#### Application of Sp1-directed site-specific ChIP on EGFR expressing tissue samples

**Figure 4:**
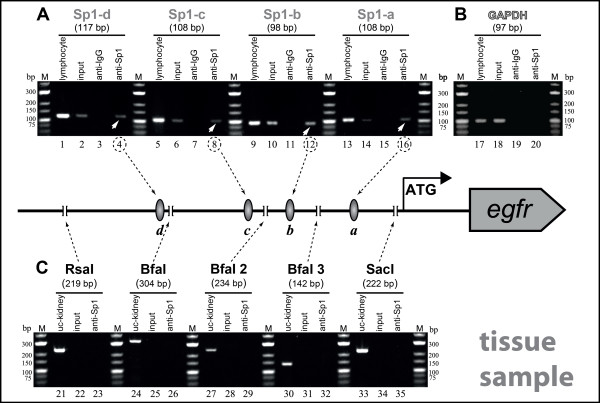
**Verification of Sp1 TFBSs targeting site-specific ChIP on tissue extracts**. Kidney carcinoma samples were chosen for adaptation of the site-specific ChIP to tissue material. Tissue samples were formaldehyde-fixed and nuclei were isolated as described. Sp1 TFBSs and enzyme cutting sites information, enzyme digestion, immunoprecipitation and PCR analysis are as described in the legend to Figure 3, with exception of the uncleaved Kidney DNA from whole cell lysate (uc-Kidney, lanes 21, 24, 27, 30, 33). White arrows depict the bound Sp1 binding sites.

The application of the modified site-specific ChIP for tissue analysis on kidney carcinoma material revealed comparable results to the experiments with cells, thereby being equally successful (Figure 4). Interactions of Sp1 with all four analyzed binding sites within the *egfr *promoter could be individually detected using the site-specific ChIP technique in combination with the Sp1-TFBSs targeting PCR (Figure 4A). The enzyme digestion control confirmed the specificity and precision of the enzymatic fragmentation (Figure 4C).

### Discussion

Even in the complex sequence environment of the *egfr*, the site-specific ChIP proved to be an adequate and effective method for the individual analysis of the TFBSs in vivo. The site-specific ChIP provides an improvement towards the standard ChIP method and further techniques for TFBS analysis. It unites the benefits of both EMSA and in vivo DNase I footprinting--the specific detection and localization of neighboring occupied binding sites in vivo--in one assay. Hence, the usually performed verification of the obtained results by a second method is therefore unnecessary (Figure 1). In combination with the enzyme digestion control by PCR using primers targeting regions spanning each enzyme cutting site, the site-specific ChIP may be generally applied for the investigation of any transcription factor recognition site along the human genome.

Completeness in a molecular sense can only be achieved by analysis methods which in situ detect all possible realised combinations of truly existing binding sites. No modern technology (including next generation sequencing) can assure that up to now. So our approach reasonably approximates this completeness. Though, in combination with a preceded consensus analysis using an adequate algorithm (like MatInspector) our site-specific ChIP is an effective method for the selective identification of complex TFBSs structures.

For all restriction sites we used restriction enzymes (BfaI, RsaI and SacI) which exhibit exactly the same digestion and buffering conditions (incubation at 37°C and 100% activity in the following restriction buffer: 20 mM Tris-acetate, 50 mM potassium acetate, 10 mM Magnesium Acetate, 1 mM Dithiothreitol pH 7.9 at 25°C). The concern that TFBSs might not be flanked by naturally occurring restriction enzyme sites and not separated by an appropriate distance from each other seems, in accordance to our experience, to be a rare event. At least in the case of the *egfr*, there were no complications.

The suitability of the used anti-Sp1-Antibody for an application in the ChIP-procedure has been checked and assured by the supplier (Merck Millipore Co., Billerica, MA). The utilized buffers are common to all antibody based ChIP assays.

By adjusting sample preparation and chromatin isolation procedures the technique is also applicable on tissue material, enabling a broad diversity of application options for clinical and molecular studies.

The site-specific ChIP is not dedicated to high throughput screening approaches but instead supports the functional analysis of a complex regulation scheme of a single gene in a systems biology view - i.g. the interaction pattern between occupied TFBSs. The focus of this work is develop a method for a) the detection of new binding sites in combination with a preceded consensus analysis and b) the individual examination of single TFBSs allocation in a complex system of neighboring binding motifs of the same type. Hence, the site-specific ChIP technique does not provide an assessment of whether the TF-binding is associated with gene transcription, what is a common limitation of all ChIP-variations. For answering the question of functional relevance further methods have to be employed. E.g. site directed mutagenesis or deletion analysis for TFBSs and promoter activity investigation *in vivo*. So, the main benefit of the site-specific ChIP lies in the investigation of specific regulatory regions in greater detail.

## Conclusions

The site-specific ChIP, which uses an endonuclease-based TFBSs specific DNA fragmentation followed by a PCR-based enzyme digestion control, opens new possibilities for the functional investigation of complex neighboring TFBSs systems of genes, even within GC-rich regions. In combination with methods for TFBSs prediction [16-18], chip-techniques and/or sequencing, it is a specific and sensitive tool for the detailed characterization of the activity of neighboring binding motifs at once. The site-specific ChIP enables the individual and reliable analysis of known and predicted binding motifs in vivo, on both cultured cells and mammalian tissue material. Hence, our method enables the detection of differences in the interaction of transcription factors and promoter regions of genes in normal and neoplastic tissues, thereby opening new possibilities for the investigation of the transcriptional regulation of genes involved in cancer biology.

## Methods

### Site-specific Chromatin immunoprecipitation

For the cell line experiments we used 10^7 ^osteosarcoma cells which were fixated by a 1% formaldehyde solution. The Nuclei were isolated using cell lysis buffer, separated by centrifugation and resuspended in adequate standard restriction buffer (New England Biolabs Inc., Ipswich, MA). Chromatin was fragmented by subjecting the nuclei to restriction enzyme digestion according to Kang et al. [19], including the following modifications. A simultaneous application of three restriction endonucleases - BfaI, RsaI and SacI (New England Biolabs Inc., Ipswich, MA) - was performed to cleave the DNA at positions flanking each Sp1 binding site within *egfr *intron 1. A complete DNA digestion was achieved by chromatin treatment with 200 U of each enzyme for 4 h at 37°C and further 100 U of each enzyme for additional 16 h at 37°C. The nuclei were then incubated with and 200 U aliquot of each enzyme and 200 U of RNase for 2 h at 37°C. Completion of restriction enzyme fragmentation was verified by electrophoresis separation on a 1.5% agarose gel. The optimization of the specific enzyme digestion based DNA fragmentation is shown on Additional file 1: Figure S1. Chromatin was isolated using nuclei lysis buffer. The lysate was diluted 10-fold in ChIP dilution buffer and equal aliquots of cleaved chromatin (equivalent to 2 million cells) from a single cell lysate were used for immunoprecipitation with antibodies against Sp1 and IgG (mock IP) as well as a control for the amount of input DNA used in precipitations (input control). 3 μg of the antibodies against Sp1 (Merck Millipore Co., Billerica, MA) and IgG (Sigma-Aldrich Co., St. Louis, MO) were used. The antibody was captured with protein A/G agarose beads (Santa Cruz Biotechnology Inc., Santa Cruz, CA). After washing the beads-antibody-chromatin complexes under stringent conditions, reverse crosslinking and purification of ChIP DNA was performed using Chelex-100 according to Nelson et al. [20]. DNA from the non-antibody whole-cell control supernatant was isolated as described in literature [20] and processed in the same way as the IP samples. All steps not mentioned here were performed according to established standards [20].

The tissues samples were processed as follows. 0.03 g frozen kidney carcinoma tissue was chopped into small pieces and thawed in freshly prepared PBS containing 1% formaldehyde for crosslinking. The tissue was homogenized to avoid cell clumps and the ChIP was performed as described above for the cell culture experiments.

### PCR based detection of immunoprecipitated TFBSs

The detection of the immunoprecipitated Sp1 binding sites was done by PCR. For each sample (DNA extracted from either the input chromatin, the normal rabbit IgG or the anti-Sp1-immunoprecipitated chromatin): 2.5 μl ChIP-DNA template, 5 pmol of Sp1 binding site specific primer, 1 U of *Taq *DNA Polymerase, 1X PCR reaction buffer II, 400 μM of each dNTP (Applied Biosystems) and 1.3 mM MgCl_2 _were mixed for PCR amplification in a 25 μl reaction volume. A defined amount of 4 ng lymphocyte DNA was used for the internal control of each PCR reaction approach. The PCR was started with an initial denaturation of 95°C for 9 min, followed by a secondary denaturation at 98°C for 1 min. Next 40 cycles of denaturation at 98°C for 10 sec, annealing at 65°C for 2 min, and a single product extension step at 72°C for 7 min followed. The PCR products were separated on a 2% agarose gel and visualized by ethidium bromide staining.

### PCR based controls of site-specific ChIP

The control of completeness of the enzyme digestion control was also performed by PCR as described and carried out on DNA templates prepared from the Sp1 immunoprecipitated chromatin sample and the whole-cell sample (input control), as well as uncleaved HOS cells DNA from the same lysate (positive control).

For the negative control, primers spanning an unrelated genomic region within the 3'-UTR of the GAPDH gene were used yielding a 97-bp product.

### Availability and requirements

Project name: Site-specific chromatin immunoprecipitation: A selective method to individually analyze neighboring transcription factor binding sites in vivo

Project home page: none

Operating systems: none

Programming language: none

Other requirements: MatInspector V7.4.8 (http://www.genomatix.de)

License: none

Any restriction to use by non-academics: none

## Abbreviations

*Egfr*: Epidermal Growth Factor Receptor gene; ChIP: chromatin immunoprecipitation; TF: transcription factor; TFBSs: transcription factor binding sites.

## Competing interests

The authors declare that they have no competing interests.

## Authors' contributions

RS, KA, BB, HB, EK contributed to the final manuscript, provided clinical samples and shared knowledge on molecular methods; AN performed experiments; RS performed experiments and developed the given method. All authors read and approved the final manuscript.

## Ethics statement

Data were analyzed anonymously. Nonetheless we have performed the study according to the principles expressed in the Declaration of Helsinki. The study was approved by the Institutional Review Board of the University of Muenster. We have acquired tissue samples only with the informed consent of the patients or patients' next of kin with the understanding by all parties that it may well be used for research. All patients provided written informed consent for the collection of samples and subsequent analysis.

## Supplementary Material

Additional file 1 **Figure S1**. Optimization of the specific enzyme digestion based DNA fragmentation. Click here for file
