# Supplementary material for: Site-specific chromatin immunoprecipitation: a selective method to individually analyze neighboring transcription factor binding sites in vivo
Source: BMC Res Notes. 2012 Feb 20;5:109. doi: 10.1186/1756-0500-5-109 (PMC3312844; doi:10.1186/1756-0500-5-109)
Supplement: Additional file 1 — Figure S1. Optimization of the specific enzyme digestion based DNA fragmentation. [file 1756-0500-5-109-S1.PDF]

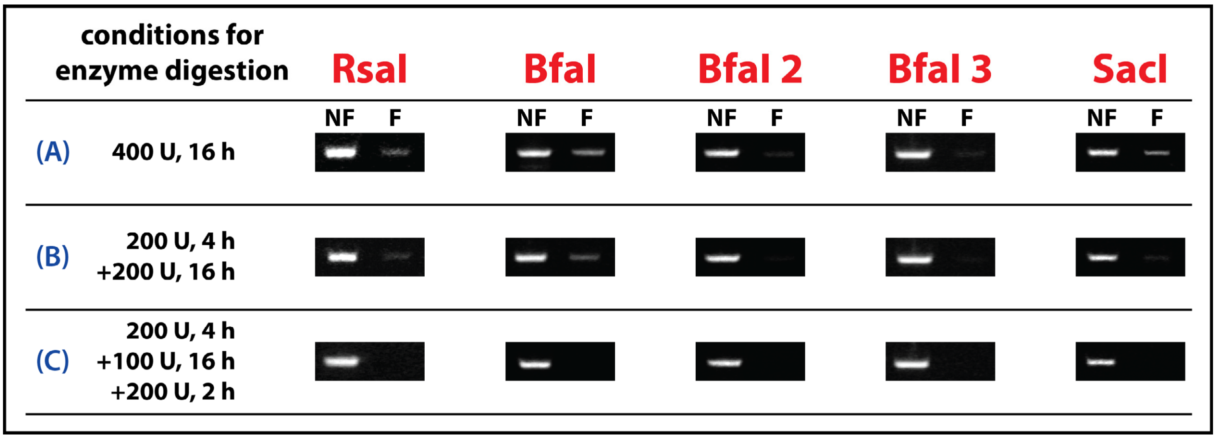

Supplemental figure 1: Optimization of the specific enzyme digestion based DNA fragmentation

Enzyme cutting sites targeting PCR analysis of the three different performed approaches for the optimization of the specific enzyme digestion based DNA fragmentation. 10 million cells were fixed with formaldehyde and subsequently lysed. The isolated chromatin was resuspended in adequate standard restriction buffer (New England Biolabs Inc., Ipswich, MA). Chromatin was fragmented by subjecting the nuclei to restriction enzyme digestion through a simultaneous application of three restriction endonucleases – BfaI, RsaI and SacI (New England Biolabs Inc., Ipswich, MA). Three different enzyme digestion conditions were tested: nuclei were treated with **(A)** 400 U of each restriction enzyme for 16 h at 37 °C; **(B)** 200 U of each enzyme for 4 h and 200 U of each enzyme for additional 16 h, both steps at 37°C; **(C)** 200 U of each enzyme for 4 h followed by further 100 U of each enzyme for 16 h and finally 200 U of each enzyme for 2 h, all steps at 37°C. Chromatin was isolated using nuclei lysis buffer, reverse crosslinked and purification of ChIP DNA was performed using Chelex-100 according to Nelson *et al.* [13]. The products of the three different digestions approaches were analysed by PCR using the enzyme cutting sites flanking primerassays (denoted in red). **NF** = not-fragmented DNA of the same lysate. **F** = fragmented DNA.
